# Stereochemical Heterogeneity Analysis of Polylactides by Multidimensional Liquid Chromatography

**DOI:** 10.1021/acs.analchem.4c00336

**Published:** 2024-03-11

**Authors:** Paul S. Eselem Bungu, Karola Luetzow, Olaf Lettau, Matthias Schulz, Axel T. Neffe, Harald Pasch

**Affiliations:** † Department of Multidimensional Polymer Characterization, Institute of Active Polymers, Helmholtz-Center Hereon, Kantstrasse 55, Teltow 14513, Germany; ‡ PSS Polymer Standards Service GmbH (Now Part of Agilent Technology), In der Dalheimer Wiese 5, Mainz 55120, Germany

## Abstract

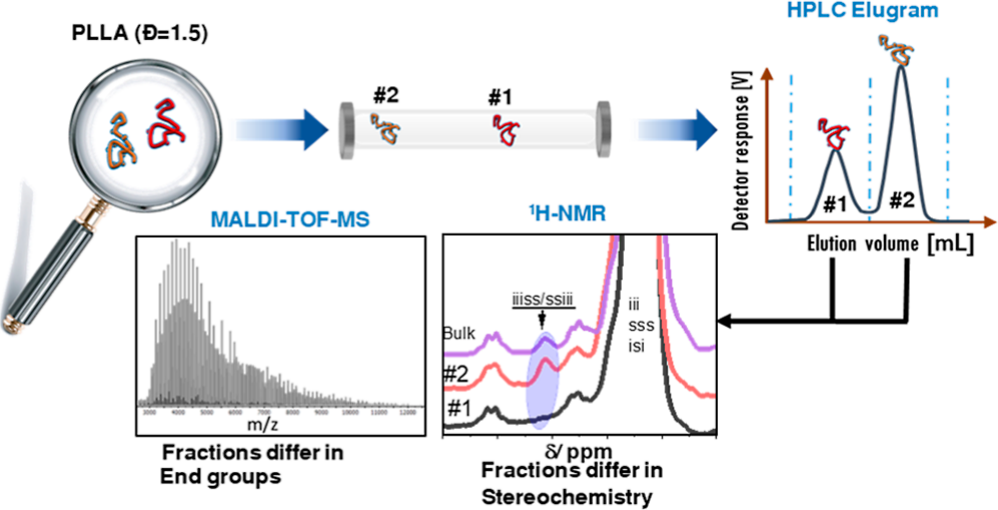

A new and robust high-performance liquid chromatography (HPLC) method that separates poly(lactic acid) (PLA) according to its stereochemical composition is presented. Using this method, poly(l-lactide) incorporating trace amounts of *meso*-lactide resulting from the racemization is separated from the pristine polymer. To prove this aspect in more detail, a representative poly(l-lactic acid) standard, assumed to be highly homogeneous, was separated using this method. The result showed that this was not the case as a fraction incorporating *meso*-lactide due to racemization occurring during the synthesis is separated. Employing two-dimensional liquid chromatography (2D-LC), the molar mass differences of the separated species were investigated, and fractions with similar molecular sizes were detected, confirming that the LC separation is solely based on stereochemical heterogeneity. The sample was further fractionated by preparative HPLC, followed by an in-depth analysis of the fractions using homonuclear decoupling in proton nuclear magnetic resonance (^1^H NMR). Convincing results that unveiled significant differences in the stereochemistry of the isolated PLA fractions were obtained. Subsequent analysis by matrix-assisted laser desorption ionization time-of-flight mass spectrometry (MALDI-TOF-MS) also confirmed oligomer series with different end group structures, indicating that the applied HPLC method is very sensitive to minor variations in stereochemistry and end groups. This integrated approach offers detailed insight into the structural characteristics of PLA polymers, contributing to a better understanding of their composition and potential applications.

## Introduction

Aliphatic polyesters, such as poly(lactic acid) (PLA), are continuously gaining interest in biomedical applications, thanks to their ability to degrade into nontoxic metabolites (CO_2_ and H_2_O). These classes of polymers are also gaining interest in academic and industrial research due to their eco-friendly properties, making them a potential replacement for traditional petroleum-based plastics used in applications such as packaging, medical implants, and drug delivery systems.^1−23^

**Figure 1:**
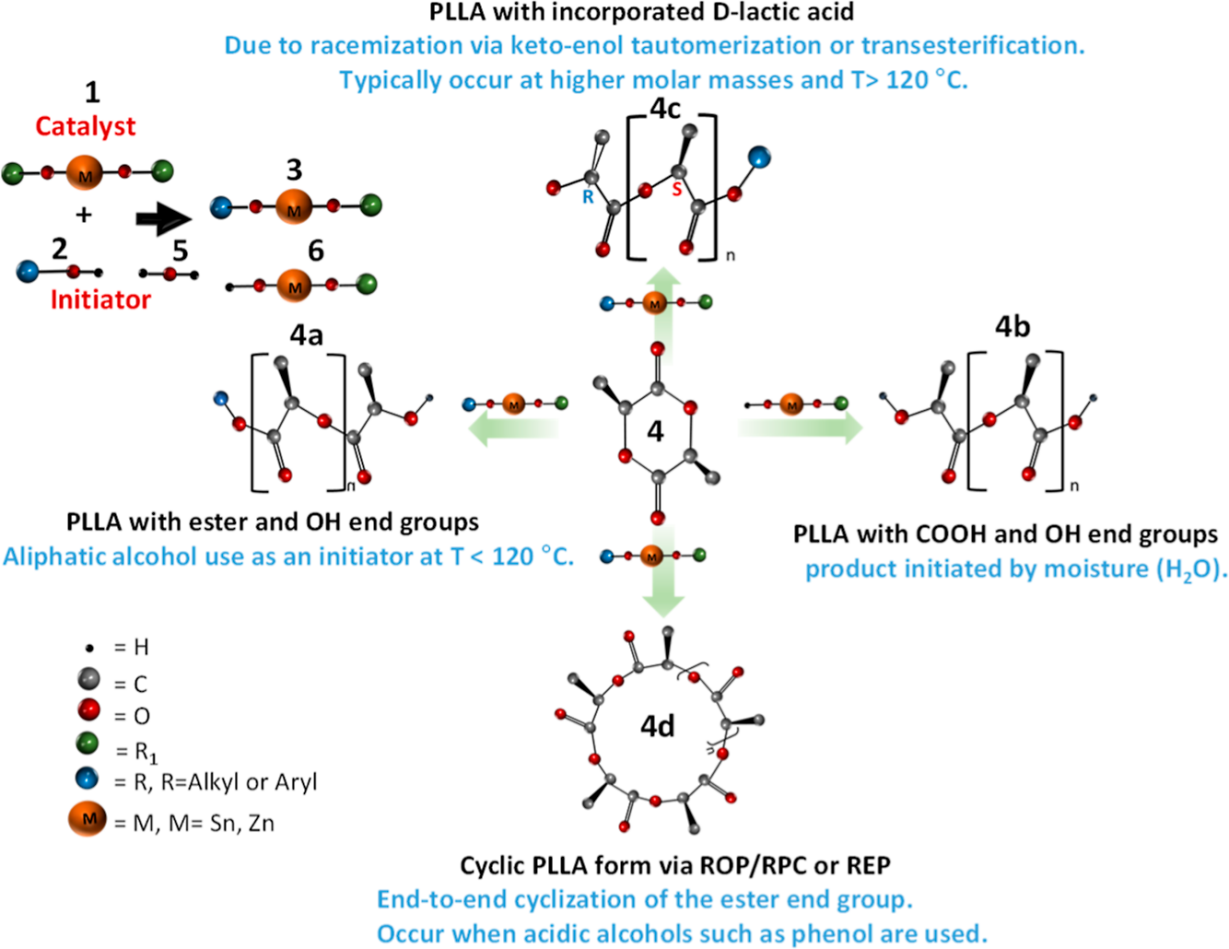
Scheme describing possible synthetic pathways in lactide polymerization. The hydrogens within the chains are omitted for better visibility.

Lactic acid (LA), the repeating unit of PLA, is derived from fermentation of biobased feedstocks, such as cornstarch and sugar cane. The chemical heterogeneity of PLA originates from the synthesis, as highlighted in the scheme in Figure 1. Commercially, PLA is typically produced *via* ring-opening polymerization (ROP) of the cyclic LA dimer, referred to as lactide, which exists in three enantiomeric forms: **l**-lactide, **d**-lactide, and *meso*-lactide. For the polymerization to occur, catalyst **(1)** (typically, tin-(II)-octanoate, Sn(oct)_2_) is first activated using alcohol **(2)** or other hydroxide-containing molecule. The polymerization proceeds *via* a nucleophilic attack by the activated catalyst **(3)** on the monomer **(4)**, followed by coordination insertion to produce polymer chains incorporating ester as end groups (–OR) **(4a)**,^4,5^ where R = alkyl or aryl from the initiator. ROP is often conducted in the melt and requires inert conditions and temperatures above 120 °C. In addition, the nonactive catalyst **(1)** can also be activated by water. Therefore, side reactions may occur in moisture **(5)**, producing PLA chains with acid end groups (COOH) **(4b)**.^6^ As the polymerization proceeds, the melt viscosity increases due to the increasing molar mass. This causes a decline in the polymerization rate as the monomer concentration decreases and becomes less accessible. This effect is counteracted by increasing the reaction temperature. However, this temperature change may also lead to racemization *via* keto–enol tautomerization of **l**- or d-lactide, forming the *meso*-lactide species. *In situ*, incorporating the *meso*-lactide produces PLA chains with decreasing enantiopurity, as indicated by product **(4c).**(7)

Besides affecting the physical and thermomechanical properties, the ratio of incorporated L and D stereoisomers significantly impacts the crystallinity and, thus, the mechanical properties and the degradation behavior of PLA.^8,9^ PLA produced from enantiopure **l**- or d-lactide forms isotactic structures that are semicrystalline, rigid, and melt at temperatures above 170 °C, making them less susceptible to hydrolytic degradation. In contrast, poly(*rac-*lactide), synthesized from a 1:1 mixture of d- and l- lactide (*rac*-lactide), is amorphous and exhibits low stiffness. This means poly(*rac*-lactide) is more prone to hydrolytic attack and exhibits faster biodegradability than that of semicrystalline polylactides. Polylactides with different thermomechanical properties are designed by varying the d and l-lactide composition.^9^

Therefore, it is crucial to accurately determine the stereochemical composition (SCC) of these complex polymers to understand the behavior and optimize the properties. This is especially important as, during the polymerization process, the stereochemistry and sequence structure of the subunits may be affected due to secondary reactions such as racemization^7,11^ and transesterification.^11^

Several existing analytical techniques, such as nuclear magnetic resonance spectroscopy (NMR),^12^ matrix-assisted laser desorption ionization time-of-flight mass spectrometry (MALDI-TOF-MS),^11^ and differential scanning calorimetry,^13,14^ have been used to address the SCC, sequence distribution, crystallization kinetics, and melting behavior of PLA. Intensive studies on PLA microstructure *via* homonuclear proton decoupling experiments have been reported. Most studies predict the stereosequence distributions and peak assignments using the Bernoullian statistics for random pairwise addition^11,15^ in combination with experimental data from the heteronuclear correlation (HETCOR).^16,17^ Slight deviations in the stereosequence distribution from the expected Bernoullian statistics were revealed to be influenced by (1) the lactide feed composition, (2) the use of initiator, (3) polymerization kinetics, and (4) the extent of conversion.^16^ Additionally, extensive MALDI-TOF investigations reported heterogeneous compositions and identified polymer chains with linear and cyclic structures from PLA produced by tin-catalyzed ROP.^10,18^ Weidner *et al.* studied the effect of using different initiators, mainly alcohols, on transesterification.^18^ Secondary reactions, such as transesterification^10,18^ and racemization,^10^ may introduce different isomers while affecting the unit patterns in the polymer backbone, as indicated by product **(4c)**.^7^ Weidner and Kricheldorf used acidic initiators such as phenol*.* and observed ROP/reversible polycondensation reaction (ROPRPC), which formed predominantly cyclic PLAs **(4d)**.^19^ Additionally, cyclic oligomers and polymers were achieved *via* ring expansion polymerization in a small amount of chlorobenzene using 2-stanna-1.3-dioxa-4,5,6,7-dibenzazepine [SnBiph] as a catalyst.^20^

The applied characterization techniques provided average structural characteristics rather than their distributions. In order to evaluate both the SCC and compositional distribution of these complex polymers after synthesis, processing, and over time, efficient and accurate methods are of high relevance.

High-performance liquid chromatography (HPLC) separates and quantifies different components and monitors structural modifications in complex materials. Barqawi *et al.*(21) applied liquid chromatography at critical conditions (LCCC) to evaluate the end group effect on aliphatic polyesters. At the same time, the separation of linear PLA from the star structures was achieved by Radke *et al.*(22) and others^17^ with LCCC. They were also able to distinguish star polymers with different numbers of arms. Li *et al.*(13) first developed an interaction chromatography method to evaluate the SCC of polylactide. In their effort, they combined LC with NMR and optical rotation and could separate enantiopure linear PLAs from chains with higher stereochemical heterogeneity based on solubility in selected solvent systems. Still, their method could not distinguish PLA chains with varying SCCs and polymer chains with trace amounts of defect. In industrial applications, gas chromatography coupled to a flame ionization detector is used after pyrolysis to evaluate the d-lactide composition in various PLAs.^4^ Feng and co-workers could likewise determine lactide compositions by HPLC after hydrolysis.^23^ Again, this method can provide only average lactide composition and not their distributions, and hydrolysis and pyrolysis may be accompanied by racemization.

Our motivation for this study was to develop and validate a suitable HPLC method that can separate PLA chains based on their stereochemical heterogeneity with high selectivity. Selected PLA standards with well-defined stereochemistry will be analyzed. Online hyphenation of this method with size-exclusion chromatography (SEC) *via* 2D-LC would determine the size distributions of the separated species. *Via* preparative HPLC coupled to homonuclear decoupled ^1^H NMR and MALDI-TOF-MS, the stereochemical differences and end groups of the separated species will be determined. Due to the high selectivity of this method, this study may be, in the end, proof that the reported stereochemistry of the selected PLA standards may not be the actual stereochemistry, thus allowing accurate quantification of **l**- and d-lactide contents and their distributions in PLA samples, enabling excellent quality control of PLA products and characterization over their lifetime.

## Experimental Section

### Materials and Methods

The PLA standards used in this investigation were purchased from PSS Polymer Standards Service GmbH, now part of Agilent Technologies (Mainz, Germany). The molar mass information of all the standards based on universal calibration and the putative stereochemistry defined by the supplier is provided in Table 1. The eluents, amylene-stabilized chloroform (TCM) and hexane (HEX) were purchased from Sigma-Aldrich. The isopropanol, tetrahydrofuran (THF), and ethanol-stabilized TCM were obtained from Fisher Scientific, TH. GEYER, and VWR International, respectively, and were all HPLC grade and were used as received. For NMR analysis, deuterated chloroform (Deutero GmbH) was used, while the *trans*-2-[3-(4-*tert*-butylphenyl)-2-methyl-2-propenylidene] malononitrile (DCTB) and sodium trifluoroacetate (NaTFA) used as the matrix and ionizing agent in MALDI experiments, respectively, were purchased from Sigma-Aldrich. MS calibration standards PMMA fleXstandard were obtained from Bruker Daltonics and SpheriCal Neat Protein Medium from Polymer Factory.

**Table 1:** Molar Mass Data of PLA Standards Are Determined by SEC Using Universal Calibration^b^

samples	PLA 0.5 K	PLA 1.5 K	PLA 3 K	PLA 8 K	PLA 18 K	PLA 28 K	PLA 72 K	cPLA 209 K
Stereochem^a^	PLLA	PLLA	PLLA	PLLA	PLLA	PLLA	PLLA	P*rac*-LA
*M*_w_/kg mol^–^^1^	0.93 ± 0.01	1.49 ± 0.02	3.68 ± 0.02	10.05 ± 0.05	17.91 ± 0.11	24.40 ± 0.12	53.85 ± 0.31	178.88 ± 0.62
		1.62^a^	3.18^a^	8.60^a^	17.8^a^	24.40^a^	69.00^a^	
*D̵*	1.08	1.05	1.35	1.37	1.52	3.67	1.12	2.05
end group	tetradecyl ester (C_14_–O–CO), OH^a^							COOH, OH^a^

a Information from supplier.

b Absolute molar masses from the suppliers determined by size-exclusion chromatography with multi-angle light scattering (SEC-MALS) are compared. Nomenclature refers to commercial designation, although molar mass may differ quite considerably. The detailed SEC experimental protocol analysis is provided in Section S1.1 of the Supporting Information.

### Interaction Chromatography

The gradient HPLC analyses reported here were conducted using an Agilent 1260 infinity II liquid chromatography system (Agilent Technologies, Santa Clara, CA, USA) equipped with a quaternary pump, a degasser, an autosampler, a column heating compartment, and a UV detector. In addition, an evaporative light scattering detector, an ELSD 1260 Infinity II (Agilent Technologies, Santa Clara, CA, USA), was used as the second detector. Separation was achieved using a Nucleosil 100 Å silica column (5 μm, 250 mm length, and 4.6 mm ID) produced by Macherey Nagel GmbH and Co. KG (Düren, Germany). Data recording and evaluation were achieved using WINGPC UniChrom Build 9050 Software (PSS Polymer Standards Service GmbH, Mainz, Germany). Samples were dissolved in TCM, and 10 μL of 1–1.2 mg/mL solution was injected during analysis. All measurements were performed in triplicates. The applied gradient for the separation is reported in Figure 3a. Using THF and TCM/TCM-OH, 60:40 ratio, as the eluent, a recovery of approximately 98% was obtained.

### NMR Spectroscopy

^1^H NMR spectra were recorded at 298 K on a Bruker AVANCE NEO 700 MHz spectrometer (Ettlingen, Germany) with a 5 mm TCI Prodigy cryoprobe. Samples were dissolved in deuterated chloroform. ^1^H NMR spectra were measured with a delay of 12 and 128 scans. Selective homonuclear decoupled spectra, in which the methyl protons resonating at 1.233 ppm were decoupled from the methine protons, were measured with a concentration of <7 mg·mL^–1^ and a delay of 3 and 32 scans.

## Results and Discussion

The analytical methods typically used in industry for determining **l**- and d-lactide compositions in PLA require a complete breakdown of the polymer chains by hydrolytic degradation or pyrolysis before determining the lactide content.^4,7,23^ These methods demonstrate high selectivity and determine lactide compositions ranging from 0.05 to 50 wt %. However, further improvements are necessary because the existing methods provide only average lactide compositions rather than their distribution. In addition, these methods are destructive and require the breakdown of the polymer chains to enable further analysis. Moreover, the methods do not account for any chemical changes that may occur during degradation due to harsh conditions such as racemization. The present investigation aims at developing a robust liquid chromatography method that can be applied to analyze the complete polymer chains and not the degraded samples. It can selectively separate polylactide chains according to their SCC while accounting for their SCC distribution (SCCD). This multidimensional chromatography approach is supported by homonuclear decoupled proton NMR and MALDI-TOF-MS.

**Figure 2:**
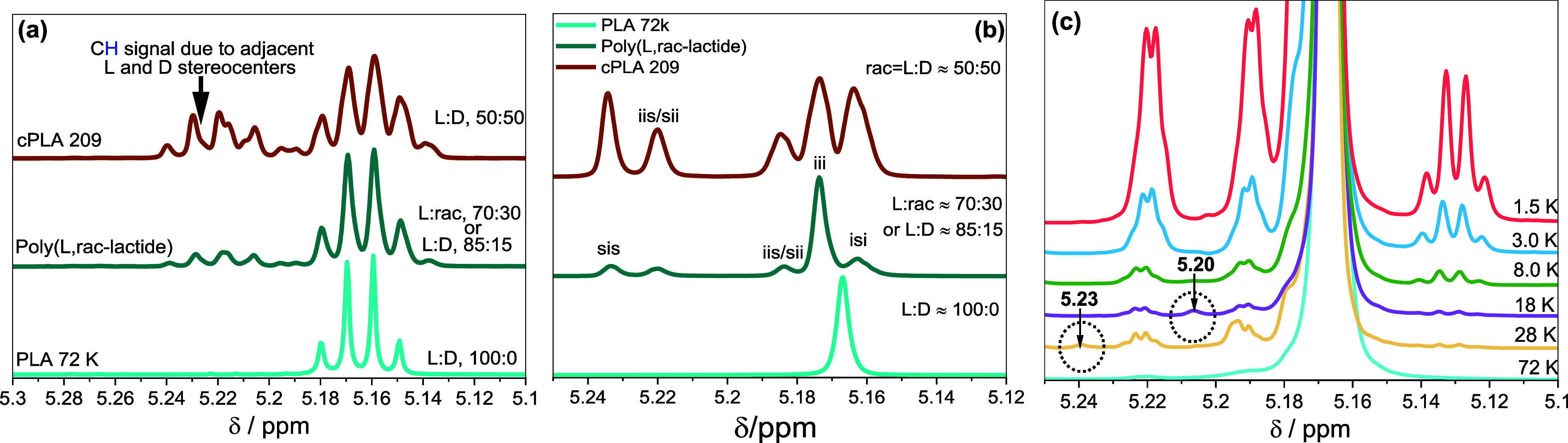
^1^H NMR spectra comparing (a) methine proton resonances and (b) homonuclear decoupled methine resonances of homopolymer, poly(**l**, rac-lactide), (70:30) or poly(**l**-/d-lactide), (85:15) and poly(rac-lactide). (c) Comparison of the homonuclear decoupled methine resonances of PLA standards.

Despite containing chiral carbons with **SS** and **RR** configurations, PLLA and PDLA enantiomers have identical NMR spectra because all atoms in the enantiomers have identical chemical environments. For example, the methine ***CH*** proton signals of PLA 72 K in Figure 2a display a quartet with chemical shifts between 5.19 and 5.14 ppm due to proton–proton coupling with the methyl ***CH***_**3**_ protons.

In poly-(*rac*-LA) synthesis, d- and l-lactides are copolymerized in a 50:50 molar ratio. According to literature findings, an alternating addition of d- and l-lactides is favored, with Sn(oct)_2_ catalyst, producing polymer chains with syndiotactic diads ([LL]_*n*_/[DD]_*m*_) or alternating SS and RR diads on their backbone.^24^ In this case, different stereosequence combinations generate different chemical environments. As shown in Figure 2a, such structural modification is highlighted by the appearance of a new set of multiplets of ***CH*** proton resonating downfield between 5.24 and 5.19 ppm for poly(**l**, *rac*-LA) and cPLA 209 K, a poly(*rac*-LA) standard. In addition, an increase in the d-lactide content from 15 mol % in poly(**l**, *rac*-LA) (70:30) to 50 mol % in cPLA 209 K also led to a corresponding increase in the peak intensities.

Due to poor signal resolution, proton–proton homonuclear decoupling was applied to simplify the spectra. Figure 2b compares the decoupled ***CH*** proton signals of all three samples. At first glance, the enantiopure PLA 72 K displays a single peak, indicating a stereosequence structure of higher homogeneity. Alternatively, similar plots of the poly(**l**, *rac*-LA) samples exhibit five peaks between 5.25 and 5.14 ppm, indicating tetrad sensitivity. This concurs well with the pairwise stereosequence distribution predicted by Bernoullian statistics for a random structure.^10^ In combination with the HETCOR correlation,^16,17^ the following stereosequences, isi, iii, sii, iis, and sis, corresponding to the respective tetrad combinations: SSRR, SSSS, SSSR, RSSS, and SRRS were assigned as shown in Figure 2b. Based on the literature, the sii and iis tetrad signals and peak probabilities are indistinguishable and are assigned interchangeably. The homodecoupled plots of the other PLA standards are compared in the zoomed-in plots in Figure 2c. Only the main peak assigned to the iii tetrads corresponding to the SSSS sequence structure is observed for most samples, indicating stereochemical homogeneity. In addition to this peak, two new peaks resonating around 5.20 and 5.23 ppm are observed for PLA 18 K and PLA 28 K, respectively (see circled peaks in Figure 2c), which correspond to the sis and ssi tetrads of poly(*meso*-LA).^7,16^ In both cases, these peaks may have resulted from the racemization effect. The peaks at 5.13, 5.19, and 5.22 ppm diminish with increasing molar mass or chain length. These peaks may be associated with the ***CH*** signals for monomer units situated very close to the ends of the chains.

The carbonyl functional group of the ester repeating units and the hydroxyl end group at the chain end render the PLA chains slightly polar and, therefore, hydrophilic. Separating these molecules by chemical composition may be possible when using a polar stationary phase in combination with good adsorbing and desorbing eluents, usually nonpolar and polar organic solvents, respectively. Li *et al.*(13) used a Nucleosil-OH (Nuc-OH) column that contains silica particles in combination with hexane and THF as the precipitating and dissolving eluent, respectively, and could separate enantiopure PLLA (SS) and PDLA (RR) from poly(*rac*-lactide) or PDLLA (SSRR) of lower enantiopurity based on their solubility differences. In addition, the homopolymers and copolymers of lower d-lactide composition coelute, irrespective of the stereochemical differences. Their observation correlated well with NMR findings.^13^ However, their method could not separate PLA chains with varying d-lactide compositions. Due to the enhanced solubility of the PLA with decreasing enantiopurity or increasing d-lactide content, THF appears too polar for such polymer systems, and therefore, no separation was observed. Finding alternative adsorbing and desorbing eluents is vital in optimizing separation based on stereoheterogeneity. TCM is an excellent dissolving solvent for most PLAs. Nevertheless, its low polarity promotes the adsorption of PLA on polar stationary phases. The adsorption-promoting effect was further enhanced by adding 70 vol % hexane to TCM, and this mixture was adopted as the adsorbing eluent. The desorbing strength of TCM was enhanced by adding 1 vol % ethanol, which increases its polarity. In this light, we introduce an alcohol-modified TCM (TCM-OH) and a 70:30 Hex/TCM-OH mixture as suitable desorbing and adsorbing eluents for this experiment.

**Figure 3:**
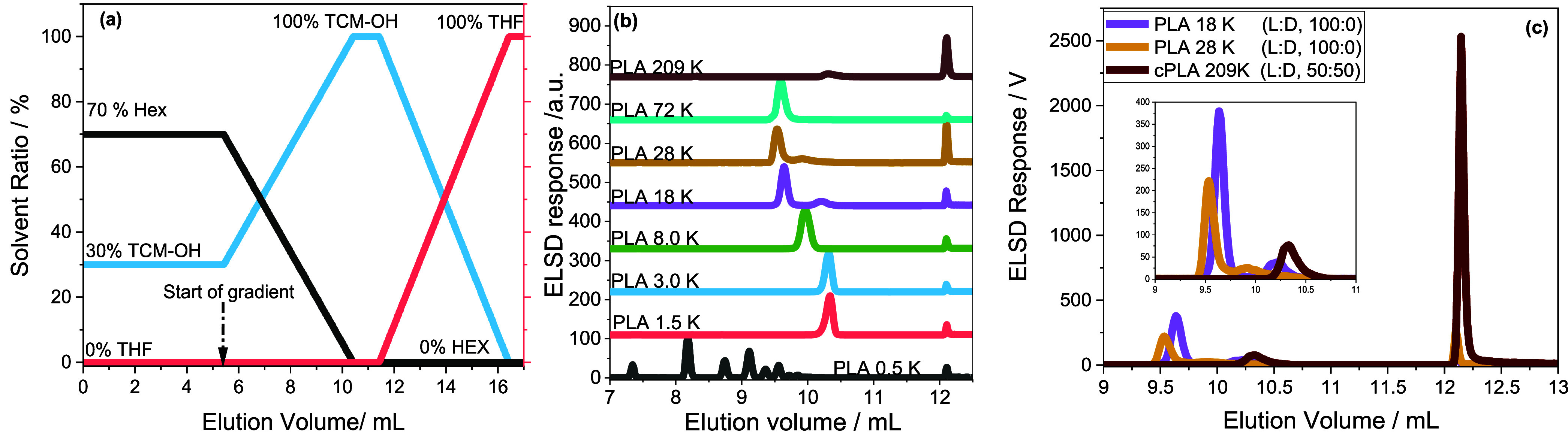
HPLC Analysis of PLA. (a) Describes the applied gradient, (b) compares the elugrams of PLA molar mass standards, and (c) compares the elugrams of samples showing more than one peak.

With this solvent system, the adsorption of PLA macromolecules on NUC-OH is promoted rather than precipitating, and the solvent system was used to develop the gradient described in Figure 3a. At the initial gradient step, the system runs isocratically at a 70:30 ratio of Hex/TCM-OH mixture (Hex: TCM-OH_0 min_) to promote adsorption on the silica. Next, a 5 min linear gradient to 100 vol % TCM-OH (TCM-OH_5 min_) is applied to desorb the polymer molecules according to increasing polarity. At the end of the gradient, TCM-OH is held isocratically for 1 min (TCM-OH_1 min_). In the last step, a second 5 min linear gradient to 100 vol % THF (THF_5 min_) is applied to wash off any strongly retained polymer from the column, such as PLA chains containing acid end groups. The separation efficiency of the method is verified by analyzing PLA standards of varying molar masses.

Figure 3b compares the elugrams of the PLA standards. Except for PLA 0.5 K, all other samples display two prominent peaks, indicating chemical composition heterogeneity. The first set of peaks elutes within the slope of the TCM-OH_5 min_ gradient, that is, between 9 and 11 mL, and the second set elutes at the start of the THF_5 min_ gradient around 12.15 mL.

For all standards containing the tetradecyl ester and hydroxyl end groups predominantly, the elution volume of the first set of peaks decreases with increasing molar mass. It becomes increasingly independent of molar masses above 20 kg/mol. This is a typical molar mass effect seen on HPLC.^25,26^ Of greater interest, the plots of PLA 18 K and PLA 28 K exhibit bimodal elution profiles with peak elution volumes of 9.64 and 10.21 mL for PLA 18 K and 9.53 and 9.91 mL for PLA 28 K, indicating compositional heterogeneity. Considering that these samples have narrow molar masses, we assume the separation is due to different chemical structures. However, the microstructural differences between the observed species still require detailed investigation, and the detailed analysis is discussed in the next section. As indicated, PLA 0.5 K displays a multimodal elugram with eight peaks between 7 and 10 mL, possibly due to PLA oligomers of varying chain lengths.

The second set of peaks consists of polymer chains eluting within the slope of the THF_5 min_ gradient, eluting around 12.15 mL, indicating stronger adsorption on Nuc-OH. Unlike the other homopolymers, cPLA 209 K is poly(*rac*-lactide) and incorporates a 50:50 molar **l**- and d-lactide ratio. The elugram of cPLA 209 K is compared with that of the enantiopure PLLAs in Figure 3b. According to the enlarged elugram in Figure 3c, cPLA 209 K exhibits two peaks: a strongly adsorbed prominent peak at 12.15 mL and a residual peak at around 10.33 mL. Interestingly, the bulk of this polymer is retained in TCM-OH but desorbs within the slope of the THF_5 min_ gradient. Furthermore, this peak appears at lower intensities in all of the samples, and their elution volume is independent of molar mass. Based on these observations, we may assume that the separation of the less retained molecules is due to hydrogen bonding interaction between the hydroxyl end groups of the polymer chains and the Nuc-OH. In contrast, the strongly retained species at around 12.15 mL of the enantiopure standard could be due to higher stereochemical heterogeneity resulting from the presence of an acid end group, as reported by the supplier.

Typically, the polar strength of molecules with protic end groups, such as primary alcohols, decreases with an increase in chain length, which correlates well with declining ***CH*** signal intensity in Figure 3c with an increase in molar mass. Therefore, the decreased elution volume with increasing molar mass can be associated with reduced polar strength. However, cPLA 209 K exhibits a more robust interaction with Nuc-OH despite exhibiting the highest molar mass. This unique elution behavior could be attributed to the stereochemical heterogeneity compared to the other homopolymers’ standard.

On the other hand, the strong retention of cPLA 209 K at 100 vol % TCM-OH is ascribed to PLA chains with low enantiopurity or high stereochemical heterogeneity and, subsequently, eluting within the TCM-OH-THF gradient. Based on this outcome, it is evident that the carbonyl groups of the repeating unit do not significantly influence the overall separation. If the backbone contributed to the separation, an increasing elution volume with molar mass would be expected due to increasing carbonyl groups. PLA 18 K was selected and fractionated by preparative HPLC to correlate the elution volume differences with the chemical structure. This sample was selected because, based on the HPLC elugram, it exhibits the highest heterogeneity and contains components mimicking the other samples.

**Figure 4:**
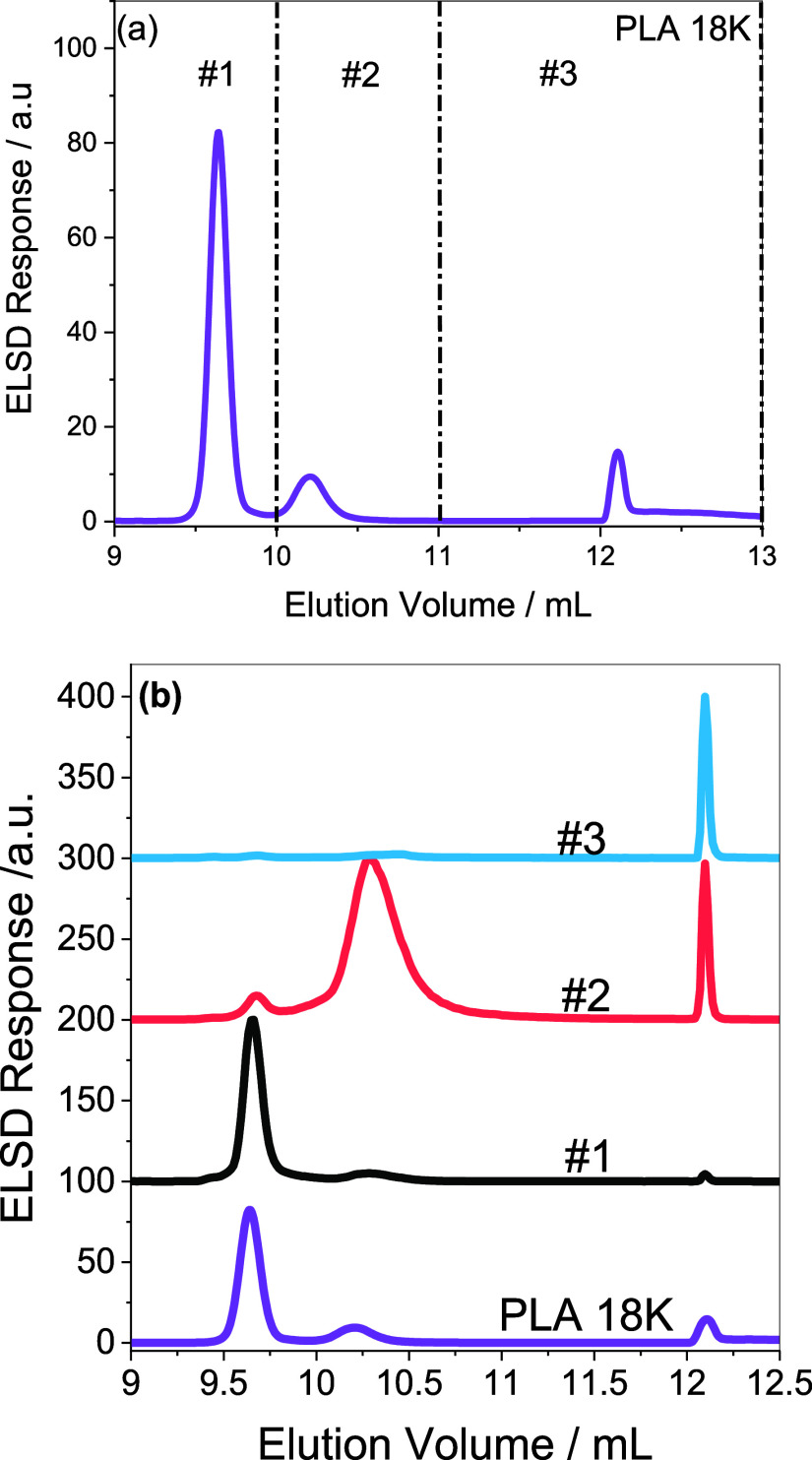
Preparative HPLC fractionation and analysis of PLA 18 K and the fractions. (a) Describes the fractionation procedure and (b) compares the elugrams of the prep fractions.

Our previous work demonstrated that preparative fractionation is the best approach to narrow the multivariate distribution of complex polymers while obtaining fractions with higher homogeneity.^27−29^ With the help of a fraction collector, three fractions were collected at different elution volumes, as highlighted in Figure 4a.

The fractions were further analyzed using the developed gradient and proton NMR. The elution profiles of the fractions are compared in Figure 4b. Accordingly, the principal peaks of fractions #1– #3 represent peaks of the targeted fractions.

Although fraction #2 elutes predominantly at approximately 10.30 mL, the fraction still contains small components of fractions #1 and #3 eluting at ca. 9.68 and 12.15 mL, respectively. At first, we could not explain this strange elution behavior of fraction #2. After a series of investigations, the coelution effect is attributed to changes in the ethanol content as different batches of commercial ethanol-stabilized TCM were used. This effect is also illustrated in Figure S1 of the Supporting Information.

**Figure 5:**
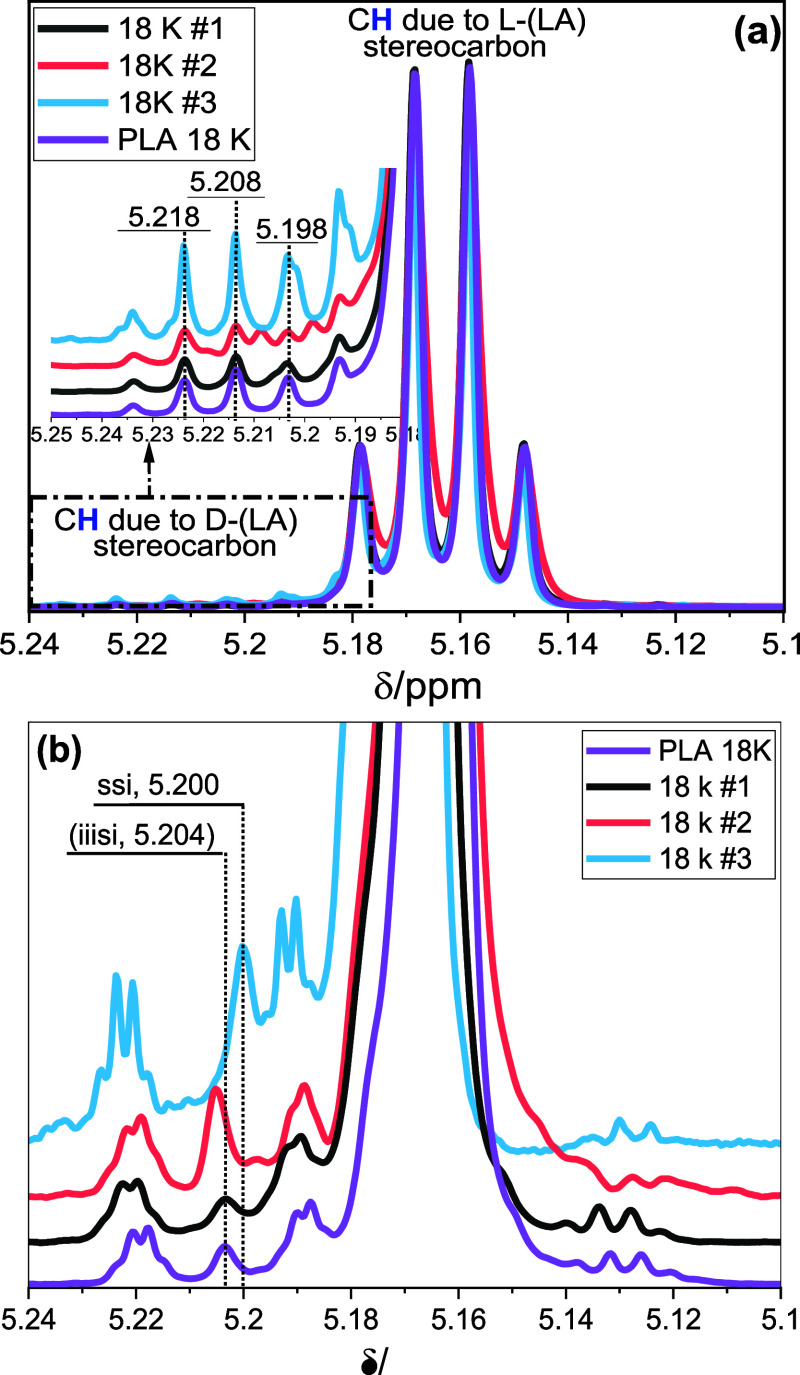
^1^H NMR spectra comparing methine proton resonance of PLA 18 K fractions: (a) coupled spectra and (b) the homonuclear decoupled spectra.

The zoomed-in ^1^H NMR spectra in Figure 5a compare the homo nuclear-coupled resonances of the methine proton of the PLA 18 K prep fractions. Here, the C***H*** resonance of fraction #1 mimics the bulk polymer since fraction #1 is the principal component of PLA 18 K. Similar peaks were seen in the spectrum of fraction #3 but with higher intensities. In comparison, the spectrum of fraction #2 shows three new peaks emerging at 5.218, 5.208, and 5.198 ppm, indicating structural diversity within the fraction. As shown in Figure 5b, applying the homonuclear decoupling experiment simplifies the complexity of the C***H*** resonances. Based on the pioneering work of Kricheldorf *et al.*(15) and others,^16^ the following conclusions were made.

First, a random stereosequence of poly(*rac*-LA) and poly(*meso*-LA) may lead to eight possible tetrads, even though some tetrad combinations may have overlapping chemical shifts. Following the Bernoullian probability, a homonuclear decoupled spectrum of poly(*rac*-LA) can only generate five tetrads, as shown in Figure 2b and the literature.^11,16,27^ In the poly(*rac*-LA) spectrum, the unresolved tetrads, sss, ssi, and iss, are only detected by ^13^C NMR. In contrast, the unresolved tetrads iis, sii, and iii of poly(*meso*-LA) can be detected in ^1^H NMR when meso lactide is present in trace amounts, usually due to impurities generated by racemization.^30^Figure 5b compares the normalized and zoomed homonuclear decoupled spectra at the methine proton region of the prep fractions. When compared with the bulk polymer, new peaks emerge. According to Thakur *et al.*,^7^ the well-resolved peak around 5.204 ppm (lit. 5.21 ppm) of fractions #1 and #2 is probably due to the iiiss hexad stereosequence with the iis tetrad core and is perhaps due to sequence structure SSSS**RS**/**SR**SSSS. Equally, the strong resonating peak at 5.200 (5.2) ppm in fraction #3 (see Figure 5b) may be due to the iissi hexad incorporating the iss/ssi tetrad core and may have resulted from the SS**SR**SS sequence structure. The possibility of the **SR** or **RS** sequence pairs is probably due to *meso*-lactide incorporation. Since no *meso*-lactide was added during the synthesis, this may have originated from the racemization effect, as previously indicated.^7,10^ From this result, it is clear that a decrease in the enantiopurity or an increase in stereochemical heterogeneity would increase the retention and, therefore, the elution volume.

**Figure 6:**
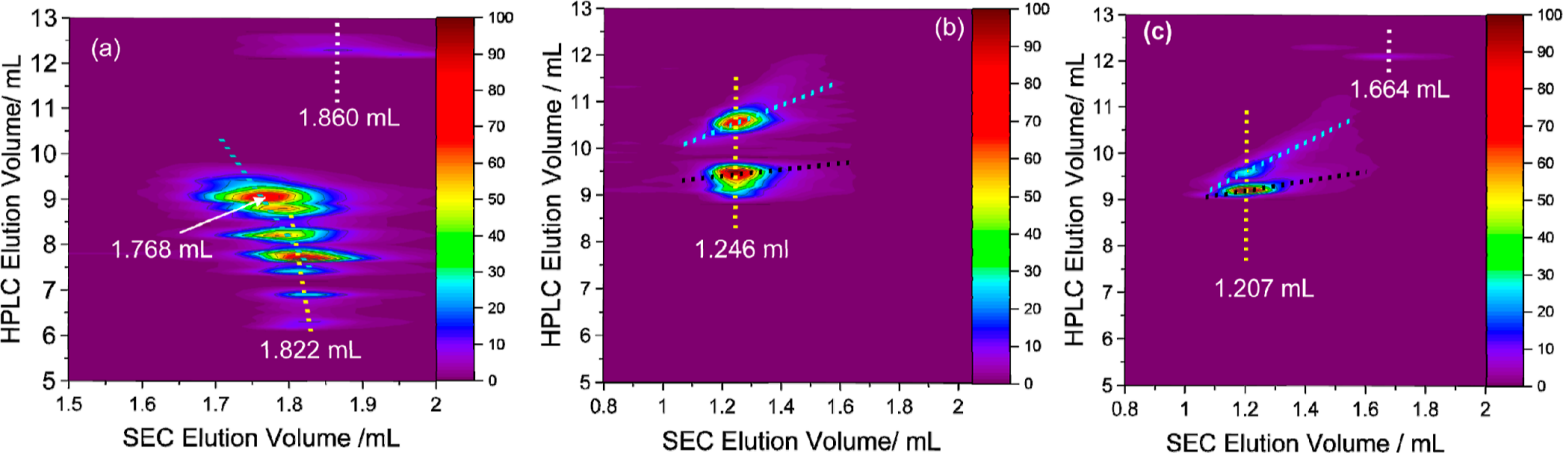
2D contour plots compare the compositional heterogeneity of (a) PLA 0.5 K, (b) PLA 18 K, and (c) PLA 28 K. Fractionations were obtained by applying the gradient in Figure 3a on the Nuc-OH column in the first dimension using a flow of 0.1 mL/min and the polarSil column in the second dimension using TCM-MeOH (5 vol %) as eluent and a flow of 1.75 mL/min.

The molar mass effect on retention was further investigated using two-dimensional liquid chromatography (2D-LC). The detailed 2D-LC experimental protocol is provided in Section S1.2 of the Supporting Information. Samples showing multimodal distributions in HPLC were analyzed further by 2D-LC. In 2D-LC, HPLC is in hyphenation with SEC, and the HPLC fractions separated based on the chemical structure in the first dimension are subsequently analyzed by SEC in the second dimension to obtain information on the molecular size distributions of the HPLC fractions. Based on the principle of SEC, this corresponds to distributions in molar mass. As previously shown, this technique provides orthogonal distribution relationships correlating chemical structure to molecular size or molar mass.^31^ The orthogonal distribution plots of PLA 0.5, 18, and 28 K are compared in Figure 6. The 2D contour plot in Figure 6a reveals multivariate distributions of PLA of 0.5 K. From the HPLC traces, eight peaks are separated between 6 and 10 mL, corresponding to the 1D analysis shown in Figure 3a. The separated species also demonstrated a decreased elution volume between 1.82 and 1.77 mL in SEC with increasing retention time in HPLC, which correlates to increased molar mass. In addition, the elution volume differences between the separated species in HPLC decrease with increasing retention volume.^32^

In contrast, two molar mass trends were observed in the SEC behavior. The first, indicated by the yellow dotted line, shows a slow increase in the molar mass for the early eluting HPLC fractions.

As expected, both polymers display bimodal distributions in the HPLC mode, indicating chemical composition heterogeneity. Still, both species coelute in SEC mode and exhibit peak elution volumes of 1.246 mL for PLA 18 K and 1.207 mL for PLA 28 K, indicating homogeneity in the molar mass (molecular size). As also indicated in black dotted lines, a slower gradient with increasing HPLC retention is seen as molar mass decreases for components with higher enantiopurity, and vice versa, a steeper slope of increasing retention for the lower enantiopure components as molar mass decreases. This agrees well with our earlier findings, where an inverse molar mass vs HPLC elution volume is seen, which may be attributed to an increase in polarity as molar mass decreases. Also, the strongly retained species eluting around 12 mL, indicated by the white dotted lines in Figure 6a,c, eluting at higher SEC elution volumes, show lower molar masses. This behavior also demonstrates fractions where chemical structure deviates from that of the principal components, and their elution volume in HPLC is independent of molar mass. These observations show that separation in HPLC is mainly due to differences in stereochemistry and end group but not molar mass, which concurs well with the NMR findings in Figure 5. At this point, stereochemistry and/or end groups play crucial roles in separating these polymer chains in HPLC.

Despite achieving such a promising HPLC separation using the TCM-ethanol gradient, the low molar mass enantiopure PLAs coelute at a higher elution volume with chains with low enantiopurity, as indicated in Figure 3b. This makes the interpretation of the results difficult. Also, our observation that the alcohol content in the commercial TCM changes may also play a key role, leading to some inconsistency in the overall elution behavior when different batches of TCM are used. In addition, the separation protocol still needs further optimization since the retention behavior is shown to be influenced by both the stereochemistry and maybe the end group and, to a certain extent, molar mass if a highly polydisperse sample is analyzed. To validate these hypotheses, it is essential to eliminate any effect caused by both molar masses and, to an extent, the OH end group on the retention volume while enhancing the separation of the polymer chains solely by their stereochemical nature and may be acid end group. In the present case, amylene-stabilized chloroform (TCMa) was used as the adsorbing eluent.

**Figure 7:**
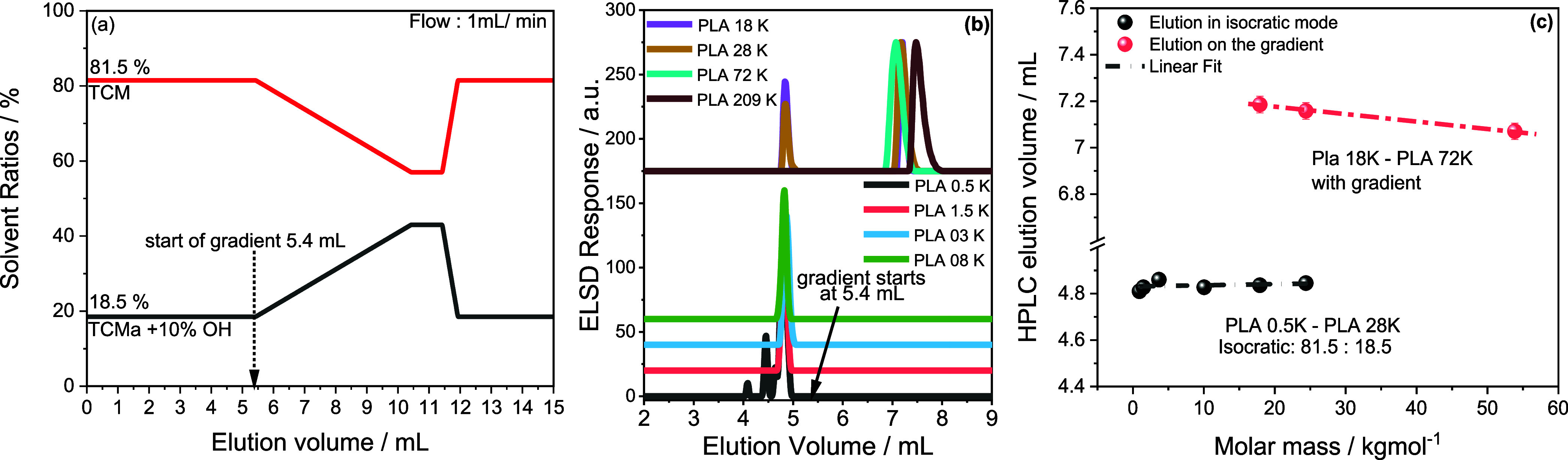
Separation of PLA standards on HPLC column. (a) Optimized HPLC gradient, (b) comparison of the elution behavior of PLA standards using this new gradient. (c) Relationship between the elution volume and molar mass of the identified peak.

For the desorbing eluent, the polarity of TCMa was enhanced by adding 10 vol % isopropanol (TCMa-OH). Usage of more alcohol aimed at eliminating any interaction due to the OH end group. Isopropanol was considered since it is a less polar solvent than ethanol. In addition, using a premixed solvent also helps eliminate any inconsistency in the alcohol content in the TCM. This solvent system is applied on the second gradient described in Figure 7a. The efficiency of the new gradient was investigated using the PLA standards. Starting with 81.5 and 18.5 vol % of TCMa and TCMa-OH eluent composition and a column temperature of 35.0 °C, we achieved the critical point of adsorption for pristine PLA chains. Elugrams from the second gradient are compared in Figure 7b. At these column conditions and in an isocratic elution, molecules of PLA 0.5 to PLA 28 K coelute at around 4.82 mL before the gradient starts at 5.4 mL.

**Figure 8:**
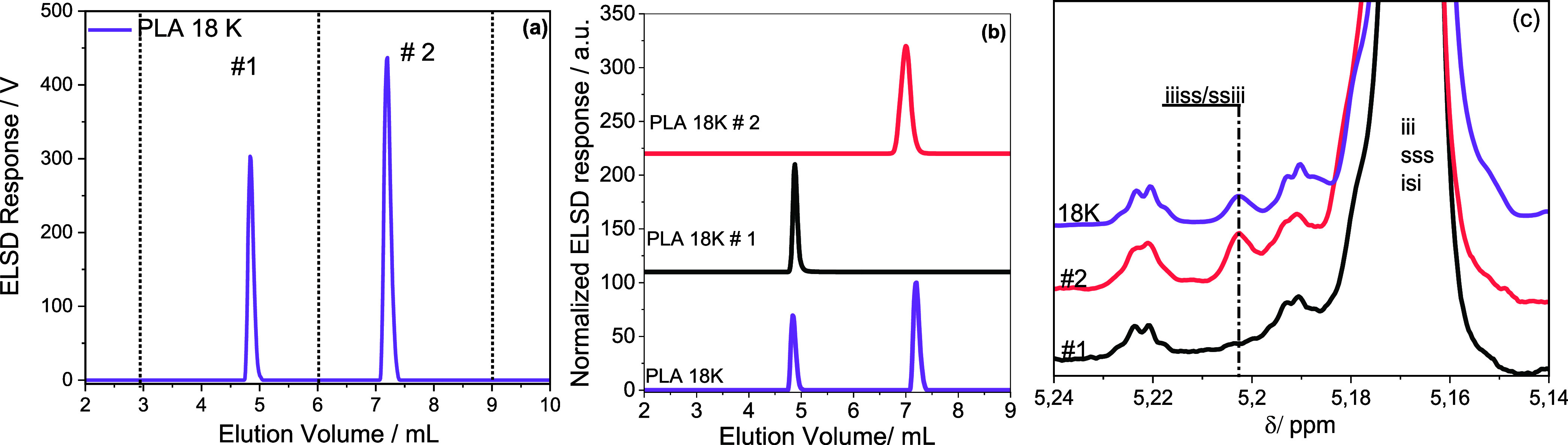
Preparative fractionation and analyses of PLA 18 K using the new gradient. (a) Prep-fractionation and (b) HPLC analysis of the fractions. (c) Homonuclear decoupled methine spectra of PLA 18 K fractions obtained from the second gradient.

A detailed description of LC at the critical point of adsorption (or critical conditions) is described in the literature.^21,22,29^ By applying a 5 min gradient to 60:40 vol %, TCMa/TCM-OH ratio, additional components of PLA 18 K and PLA 28 K are desorbed along the slope of the TCMa-OH_5 min_ gradient at 7.19 and 7.16 mL, respectively, indicating compositional heterogeneity. Interestingly, PLA 72 K and cPLA 209 K display single peaks at 7.07 and 7.49 mL, respectively. The shift to higher elution volumes may be attributed to changes in SCC, or the molecules may constitute acid end groups. The relationship between the molar mass and the HPLC elution volume of all samples is compared in Figure 7c. Accordingly, the elution volume of the components around 4.8 mL is independent of molar mass, as indicated by the black dashed line. In contrast, a slight decline in the elution volume with increasing molar mass is seen for the retained species, as shown by the red dashed line. This may be assigned to a decreasing level of stereochemical heterogeneity with increasing molar mass. To align the different components of PLA 18 K to the chemical structure, PLA 18 K was again fractionated by preparative HPLC, using the second gradient, as indicated in Figure 8a. HPLC and proton–proton homonuclear decoupled ^1^H NMR were used to analyze the collected fractions further. Figure 8b compares the elugrams of the fractions and the bulk polymer. As seen, fraction #1 constitutes mainly the less retained components at 4.8 mL, while fraction #2 comprises predominantly the retained components, with an elution volume of around 7.19 mL. The methine proton resonances of the homonuclear decoupled spectra of the fractions are compared in Figure 8c. Accordingly, the emerging peak around 5.207 ppm is due to the hexad iiiss/ssiii generated by the SSSSRS/SRSSSS sequence with the iis/sii tetrad core. As indicated earlier, this is typically due to the incorporation of *meso*-lactide impurities and may have resulted from the racemization effect. The peak resonating around 5.204 ppm in bulk is absent in fraction #1, indicating no stereochemical heterogeneity. On the other hand, the same peak appears strongly in the most retained fraction #2, indicating stereochemical heterogeneity.

**Figure 9:**
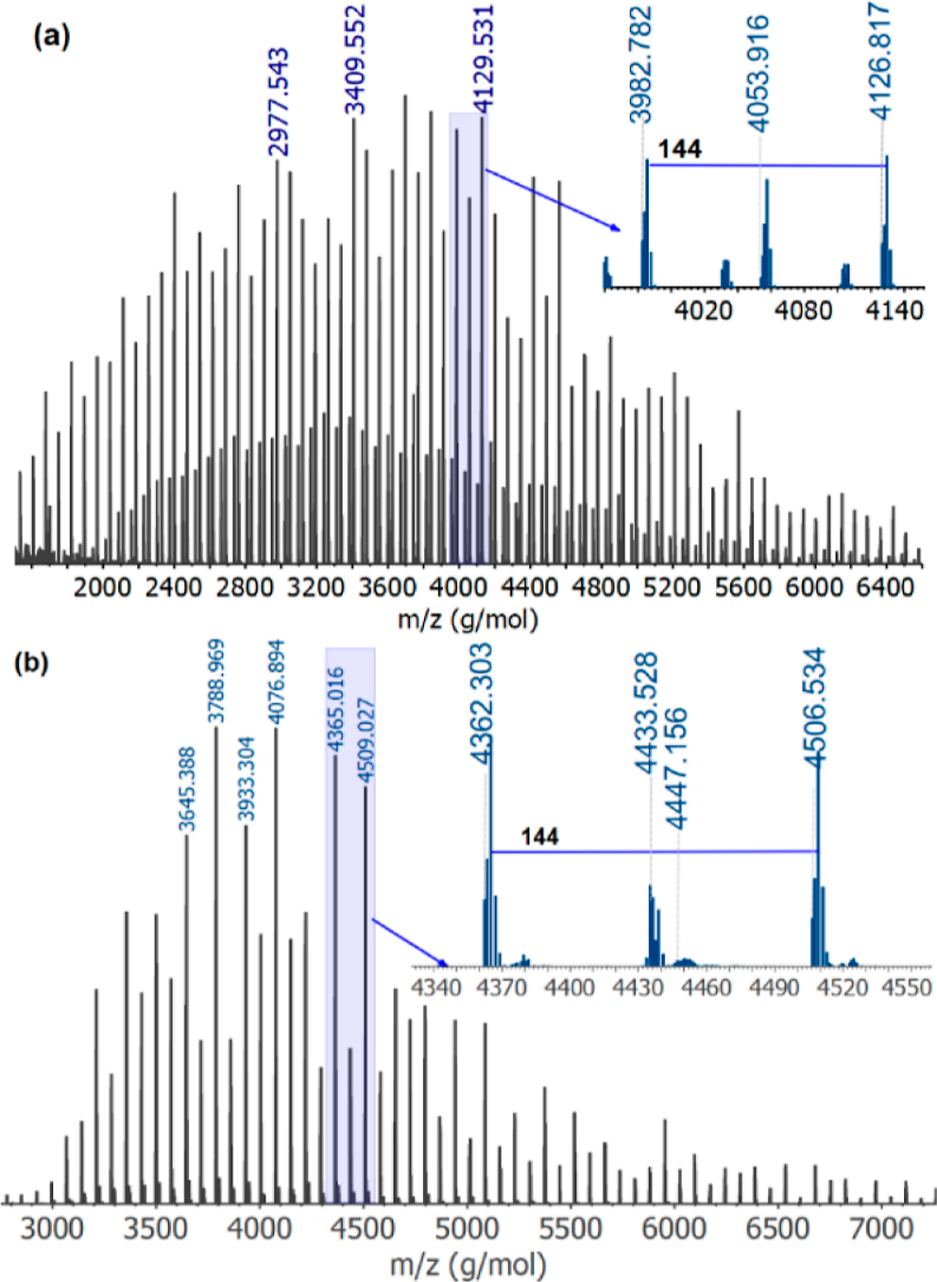
MALDI-TOF mass spectra confirming the end groups of (a) fraction 1 and (b) fraction #2. Spectra were recorded using a 1:10:1 mixture of analyte, DCTB, and NaTFA in THF.

MALDI-TOF-MS in linear and reflective modes was used to analyze the fractions to determine their end groups. The detailed MALDI-TOF-MS experimental protocol is provided in Section S1.3 of the Supporting Information. The spectra recorded in linear mode display a higher molar mass range for the fractions, as shown in Figure S2 of the Supporting Information. The MALDI-TOF mass spectra of the PLA 18 K HPLC fractions presented in Figure 9 were recorded in reflective mode and used to identify the end groups of the fractions.

The spectrum in Figure 9a was recorded from fraction #1. It displayed three different series with a characteristic peak-to-peak distance of 144 Da, as shown in the enlarged plot, typical of the lactide repeating unit. The first series, which is defined by *m*/*z* values of 3982.782 and 4126.817, represents the main series with the general formula H(O_4_C_6_H_8_)_*n*_OC_14_H_29_Na^+^, where *n* corresponds to 26 lactide repeating units bound by tetradecyl ester and hydroxyl end groups.

The second series, defined by the *m*/*z* of 4053.916, is characterized by the addition of 72 Da from *m*/*z* of the first series and corresponds to the repeating addition of lactic acid and is defined by the general formula H(O_4_C_6_H_8_)_*n*_O_2_C_3_H_4_OC_14_H_29_Na^+^. According to the recently published work by Kirchhecker *et al.*(6) and the literature therein,^11^ the peaks related to 2*n* + 1 series (lactic acid repeating units) resulting from transesterification.

The third series of fraction #1 is due to the ionization by hydrogen and has the formula H(O_4_C_6_H_8_)_26_OC_14_H_29_H^+^. Therefore, fraction 1 constitutes predominantly the tetradecanol-initiated PLA chains. A similar spectrum of fraction #2 is presented in Figure 9b. Accordingly, the mass spectrum displays three molar mass series with a peak-to-peak distance of 144 Da, as indicated in the enlarged plot. The series with *m*/*z* of 4362.303 and 4506.534 consists of lactide repeating units bound by acid and hydroxyl end groups and is defined by the formula H(O_4_C_6_H_8_)_*n*_OHNa^+^, where *n* is 30 for the peaks in the enlarged plot. The second series is due to transesterification and demonstrates a peak distance of 72 Da. These results agree with the HPLC data as the acid end makes the chains more polar due to the two polar end groups. The last and final series exhibit a peak distance of 14 Da, calculated from the prominent peaks with *m*/*z* = 4433.528 of the second series. This mass difference is due to methyl addition on the main series, which may originate from an exchange between the proton on the acid end group and the methyl from methanol forming methyl ester end group and exhibit the general formula H(O_4_C_6_H_8_)_*n*_OCH_3_Na^+^.

These species may have been formed during the termination step of the reaction as methanol is typically used as the quenching agent. Therefore, it can be concluded that racemization occurs predominantly on the acid-functionalized molecules as fraction #2 shows both acid end group and racemic characteristics as observed on MALDI-TOF and homonuclear decoupled NMR, respectively.

## Conclusions

PLA polymers are an essential class of biodegradable materials used in biomedicine and other industrial fields. Therefore, detailed analysis of these polymers in terms of their composition, addressing stereochemical and end group heterogeneity, is crucial for quality control and ensuring consistency in product design. This needs a close look at precise analytical measurements, ensuring a high selectivity in separation to unveil such heterogeneities in PLA materials. In this study, a robust HPLC method for separating PLA chains incorporating minor differences in SCC with enhanced selectivity was developed. To illustrate this, a representative sample (PLA 18 K) that exhibits bimodal HPLC elution was selected. Combining this HPLC method with homonuclear decoupled NMR spectroscopy and MALDI-TOF mass spectrometry *via* preparative fractionation proved that chains with higher stereo chemical defects were separated from those with higher stereo chemical homogeneity. The defects were shown to have resulted from the incorporation of *meso*-lactide impurities, shedding more light on specific reaction pathways and factors influencing the SCC.

For the first time, insight into the racemization effect based on end group acidities was shown, providing practical implications in optimizing PLA synthesis processes for the desired stereochemical outcome. Therefore, this work contributes to the existing body of knowledge and provides valuable insight with implications for the synthesis and characterization of PLA polymers. While this study focuses on studying PLA standards claimed to be PLLA with high homogeneity, our method could show that this was not the case due to racemization occurring during the synthesis. The method shows a good separation of samples with minor stereochemical differences. Therefore, our method can be applied in studying synthetic conditions, for use in quality control of commercial materials, and for monitoring PLA degradation characteristics over time. The methodology and findings can potentially guide future research and application in the field of polymer science and materials engineering.

## Supporting Information Available

SEC, NMR, and MALDI-TOF-MS experiments; HPLC elugrams obtained with TCM containing different ethanol concentrations; MALDI-TOF spectra of PLA 18 K fractions obtained in linear mode; and full NMR spectra of PLA 18 K fractions (PDF)
