# Supplementary material for: Stereochemical Heterogeneity Analysis of Polylactides by Multidimensional Liquid Chromatography
Source: Anal Chem. 2024 Mar 11;96(11):4716–25. doi: 10.1021/acs.analchem.4c00336 (PMC10955512; doi:10.1021/acs.analchem.4c00336)
Supplement: Supplementary file 1 — ac4c00336_si_001.pdf [file ac4c00336_si_001.pdf]

Supporting Information

Stereochemical Heterogeneity Analysis of Polylactides by Multidimensional Liquid Chromatography

Paul S. Eselem Bungu*[a]†, Karola Luetzow[a]†, Olaf Lettau[a]†, Matthias Schulz[b], Axel T. Neffe[a]† and Harald Pasch*[a]†

[a]Department of Multidimensional Polymer Characterization, Institute of Active Polymer, Helmholtz-centrum Hereon, Kanstrasse 55, 14513 Teltow, Germany

[b]PSS Polymer Standard Service GmbH (now part of Agilent technology), In der Dalheimer Wiese 5, 55120 Mainz, Germany

†Department of Correlative Characterization, Institute of Functional Materials for Sustainability, Helmholtz-center Hereon, Kanstrasse 55, 14513 Teltow, Germany

*Authors to whom Corresponding should be addressed:

Dr. Paul S. Eselem Bungu
E-mail: paul.eselem@hereon.de

Prof. Dr. Harald Pasch
E-mail: harald.pasch@hereon.de
https://www.hereon.de/

Table of Content

1 Supplementary Experimental Section....................S-3
1.1 Size Exclusion Chromatography (SEC)....................S-3
1.2 Two-Dimensional Liquid Chromatography (2D-LC):....................S-3
1.3 Matrix-Assisted Laser Desorption Ionization Time-Of-Flight Mass Spectrometry (MALDO-TOF-MS): S-3
2 Supplementary Result and Discussion....................S-4
Figure S1 HPLC Elugram comparing different ethanol Concentrations....................S-4
Figure S2 MALDI-TOG-MS spectra obtained in linear Mode. S-4
Figure S3 NMR Spectra of PLA Fractions S-4

1 Supplementary Experimental Section

1.1 Size Exclusion Chromatography (SEC)

The molar mass and molar mass dispersity measurements were carried out using the Agilent 1260 Infinity II GPC system (Polymer Standards Service GmbH, Mainz, Germany) equipped with an isocratic pump, a degasser, an autosampler, a column-heating compartment, a UV and a RI detector. Additionally, PSS SLD7000 MALS (Polymer Standards Service GmbH, Mainz, Germany) and PSS DVD1260 online viscometer (Polymer Standards Service GmbH, Mainz, Germany) detectors were used to determine absolute molar masses based on lighter scattering measurements and universal calibration. Polymer separation was achieved using a Lux guard column, 10 µm, 50 mm × 8 mm ID and two SDV 10 µm, 300 mm × 8.0 mm ID analytical, linear XL columns (Polymer Standards Service GmbH, Mainz, Germany). Polymer molar masses were evaluated with the help of WinGPC UniChrom software version 8.3 Build 9050 (Polymer Standards Service GmbH, Mainz, Germany, PSS) using a universal calibration approach, which was obtained by applying polystyrene standards with Mn between 580 g·mol−1 and 975 000 g·mol−1 (Polymer Standards Service GmbH, Mainz, Germany). The eluent, TCM, was stabilized with ethanol (0.6 – 1 vol%) and used at a 1 mL/min flow. All samples (4 mg) were dissolved using TCM (1 mL) containing toluene (0.1 vol%) overnight, and 50 µL of the solution was injected. The toluene peak was used as the flow rate maker for all analyzes. Molar masses were determined by GPC via intrinsic viscosity using universal calibration, obviating the need for prior determination of dn/dc. All measurements were conducted in triplicates.

1.2 Two-Dimensional Liquid Chromatography (2D-LC):

Two-dimensional chromatography separations were performed by coupling the IC and SEC using a valve system (VICI Valco instrument, Houston, Texas, USA) controlled by the WinGPC software and equipped with two 100 µL sample loops. The samples (4 mg) were dissolved in TCM (1mL), and a 100 µL solution was injected into the first dimension column using the described gradient in Figure 3a but applying a flow rate of 0.1 mL/min. In the second dimension, all injected samples were fractionated using PolarSil 300 Å (3 µL, 150 mm length and 4.6 i.d.) produced by Polymer Standards Service GmbH, Mainz, Germany. The column was placed in an external holder at room temperature, with a 2 mL/min mobile phase flow rate. Data were collected using an Agilent 1290 infinity II ELSD (G7102A) and processed using WinGPC UniChrom software version 8.4 Build 9999 (Polymer Standards Service GmbH, Mainz, Germany, PSS).

1.3 Matrix-Assisted Laser Desorption Ionization Time-Of-Flight Mass Spectrometry (MALDO-TOF-MS):

MALDI-TOF mass spectra were recorded on a BrukerUltraflex (Bruker Daltonics, Bremen, Germany) in the linear and reflective mode. The samples (5 mg·mL-1), the matrix, DCTB (20 mg·mL-1), and the ionizing agent, NaTFA (13 mg·mL-1), were dissolved in THF and were mixed in the ratio 1:10:1. 1 µL of this mixture was spread on spots on a MALDI ground steel target plate. Spectra were recorded and evaluated with the manufacturer's software (FlexControl, FlexAnalysis, PolyTools). Typically, 1000 shots on different positions were accumulated. The spectra were calibrated below 9 kDa with PMMA (fleXstandard, Bruker Daltonics, Bremen, Germany) and above 9 kDa with SpheriCal® Neat Protein Medium (Polymer Factory, Stockholm, Sweden). All measurement were conducted in triplicates

2 Supplementary Results and Discussions

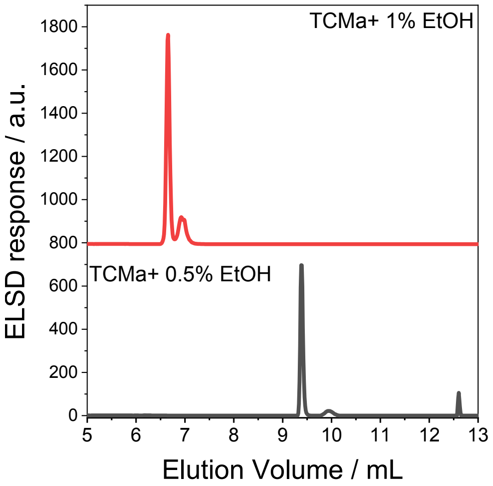

Figure S1: **HPLC Elugram Comparing Different Ethanol Concentrations**
HPLC elugrams comparing the elution profiles of PLA 18K using different ethanol content in TCM. In addition to observing peaks shift to lower elution volumes with increasing ethanol content, the separation margin between the two species also decreases. At lower ethanol content, a third peak is observed around 12.6 mL when introducing THF. At higher ethanol content, the peak disappears. This elution behavior shows that the 0.5 vol% ethanol was insufficient to desorb all the retained molecules.

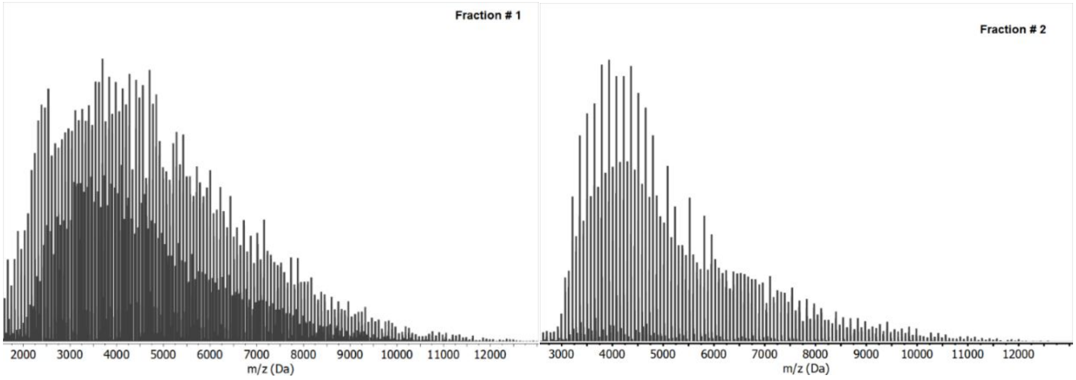

Figure S2: **MALDI-TOG-MS spectra Obtained in Linear Mode.**
MALDI-TOF mass spectra of (a) Fraction #1 and (b) Fraction #2. Spectra were recorded in linear mode using a 1:10:1 mixture of DCTB, analyte, and NaTFA in THF.

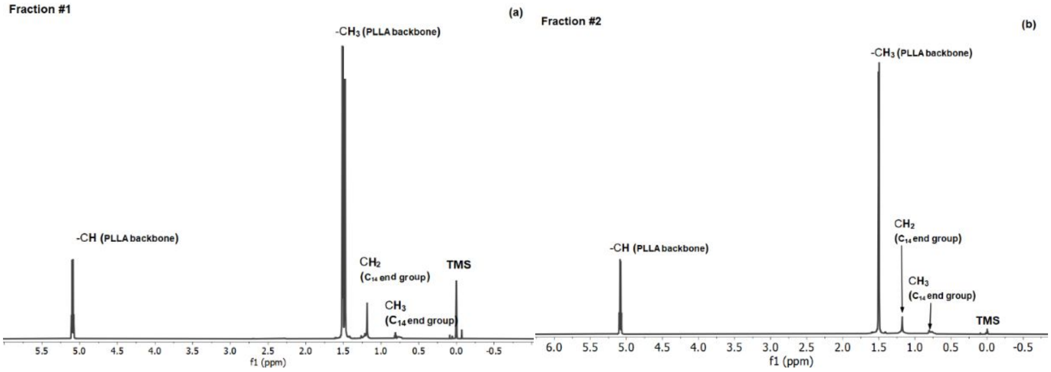

Figure S3: **NMR Spectra of PLA Fractions**
1H-NMR homonuclear coupling spectra of PLA 18K Fraction 1 and 2. Spectra were recorded after a successful preparative HPLC of PLA 18K.
